# Surgical placement possibilities, audiological benefit, and quality of life following Bonebridge implantation in children

**DOI:** 10.1371/journal.pone.0332978

**Published:** 2025-10-09

**Authors:** Milan Urík, Soňa Šikolová, Vít Kruntorád, Dagmar Hošnová, Jan Šíma

**Affiliations:** 1 Department of Pediatric Otorhinolaryngology, University Hospital Brno, Brno, Czech Republic; 2 Faculty of Medicine, Masaryk University Brno, Brno, Czech Republic; LSU Health Shreveport, UNITED STATES OF AMERICA

## Abstract

This study’s primary goal was to evaluate how smaller size of the BCI602 transcutaneous bone conduction implant affects surgical placement possibilities in children compared to its precursor model, the BCI601. Additionally, audiological benefits, quality of life, and surgical safety of the implant system were investigated in a retrospective cohort of 21 patients. Computed tomography imaging data of the previously implanted patients were examined using a surgical planning software to determine which locations on the scull were feasible for placing the floating mass transducer of the respective implant version. Audiological outcomes in terms of sound field audiometry, speech recognition threshold, and speech recognition in noise were assessed. The Speech, Spatial, and Qualities of Hearing Scale (SSQ12) and the Assessment of Quality of Life 6 Dimensions (AQoL-6D) questionnaires were used to assess subjective benefit in terms of hearing and quality of life. Evaluation of placement options revealed that placement of the BCI602 in the transmastoid plane was possible in 100% of the children, as well as in 89.5% of the cases with the BCI601 variant when using lifts. Likewise, it would be possible to place the BCI602 in 100% of patients superior to the temporal line. For the older BCI601 version, this would be possible only in 58% of cases. With the implant, audiological performance as well as subjective benefit scores improved significantly in the study population. The pediatric cohort reports significant audiological benefit, significantly improved quality of life, and low complication rates. Its combination of high safety, significant audiological benefits, and easy placement of the BCI602 makes the Bonebridge a comfortable and effective option for hearing rehabilitation in children.

## Introduction

The Bonebridge hearing implant (MED-EL, Innsbruck, Austria) is an active transcutaneous bone conduction implant (BCI) suitable for various types of hearing loss and is indicated for subjects 5 years of age and older. Bone conduction implants have particularly benefited people with mild to moderate conductive hearing loss (CHL) and mixed hearing loss with bone conduction thresholds ≥45 decibels hearing level (dB HL). The implants also constitute an option for patients suffering from single-sided-deafness (SSD). The importance of recovering hearing loss in the pediatric population as quickly as possible is widely accepted and has been studied for all kinds of hearing rehabilitation devices. The first-generation Bonebridge, the BCI601, has been the subject of numerous studies, and a systematic review has shown the significant and stable benefit of the device as well as its long-term safety in children and adults [1]. One reported drawback of the BCI601, consisting in the size of the implanted floating mass transducer (FMT), has been addressed by the manufacturer in its latest generation of the device, the BCI602. Compared to the previous version, the new implant requires nearly 50% less drilling depth, because the height of the FMT has been reduced from 8.7 mm to 4.5 mm. The required depth of the BCI602 implant bed is almost equal to the drilling depth of a bone-anchored hearing aid (BAHA) screw without the necessity for osteointegration. This reduction in implant height, and hence drilling depth, is a considerable advantage and creates opportunities for its use in patients for whom to date the first-generation implant could not be used due to limitations of cortical thickness. The BCI602 is reported to meet the same audiological and medical criteria as did the first-generation device [2–5]. Especially in pediatric patients, a safe surgical procedure is the most crucial aspect when choosing an implantable device to improve hearing even as its effectiveness, too, is very important. Early data indicate that the BCI602 allows for a safe procedure as well as good audiological outcomes in children.

The Bonebridge device is composed of an external audio-processor and the FMT placed transcutaneously into the temporal bone. Depending on anatomical conditions, the FMT might be placed in one of various positions in the temporal bone and can be bent at its transition in both horizontal and vertical planes. The retrosigmoid or middle fossa approach can be used in cases where either the sigmoid sinus is too anterior, the dura mater in the middle cranial fossa is too low, or there has previously been a mastoidectomy. Independent of surgical approach, exact placement requires avoidance of the aforementioned critical structures. That means careful evaluation of the temporal bone contour and thickness is mandatory [6]. More importantly, such critical structures as the sigmoid sinus or underlying dura should be avoided, as sigmoid sinus injury can lead to air bleeding, embolisms, thrombosis, discomfort, pain, leakage of cerebrospinal fluid, or hematoma. In the case of the first-generation BCI601, preoperative planning software enabled surgeons accurately to determine ideal FMT positioning while avoiding potential injuries and potentially reducing surgery times. Even though preoperative planning is only optional in relation to the new generation BCI602, this study aimed to show how frequently and under what circumstances placement of either device would have been possible. A study by Vyskocil et al. had investigated patients who received additional non-osseous stimulation via the dura and/or sinus; it showed no statistically significant audiological differences compared to a group with exclusively bone conduction stimulation [7]. Furthermore, a possible beneficial positive effect of soft-tissue conduction or non-osseous bone conduction as well as bone conduction (hybrid stimulation tested in humans and cadavers) is still debated [8–10]. OTOPLAN is a software for otologic surgical planning developed by CASCINATION (Bern, Switzerland) in cooperation with MED-EL (Innsbruck, Austria). Most notably, it is used for preoperative planning of cochlear implant surgeries, but OTOPLAN also can be used to estimate skull thickness and possible FMT placement for Bonebridge implants. The software uses a patient’s preoperative computed tomography (CT) images following what is known as temporal bone protocol to investigate the placement possibilities and to plan FMT positioning. Surgeons can preoperatively determine possible dura or sinus involvement and necessity for using implant lifts.

The primary aim of this study was to evaluate FMT placement possibilities of the two generations, the BCI601 and BCI602. We investigated the different implant placement options in our dataset, which is to say how often classical transmastoidal (also known as sinodural angle placement), retrosigmoidal implantation, and superior to temporal line positioning could be used in surgery. In addition, we assessed whether the placement would require lifts and/or required a dura and sinus involvement. Audiological benefit, quality of life (QoL), and implant safety also were evaluated. To the best of the authors’ knowledge, this is the first study investigating surgical placement options together with audiological and QoL measures in a pediatric population.

## Materials and methods

### Study population

The prospective data analysis and implantation were performed as part of routine clinical procedures at a tertiary center between December 2020 and July 2024. The study protocol was approved by the ethics committee of University Hospital (No. 03–041120) and informed consent of the parents or legal guardian was obtained prior to surgical intervention. The audiological inclusion criteria were based on the manufacturer’s recommendations and pediatric patients suffering from mixed and/or conductive hearing loss and SSD were included.

### Surgical planning software: Implant placement evaluation

**Fig 1:**
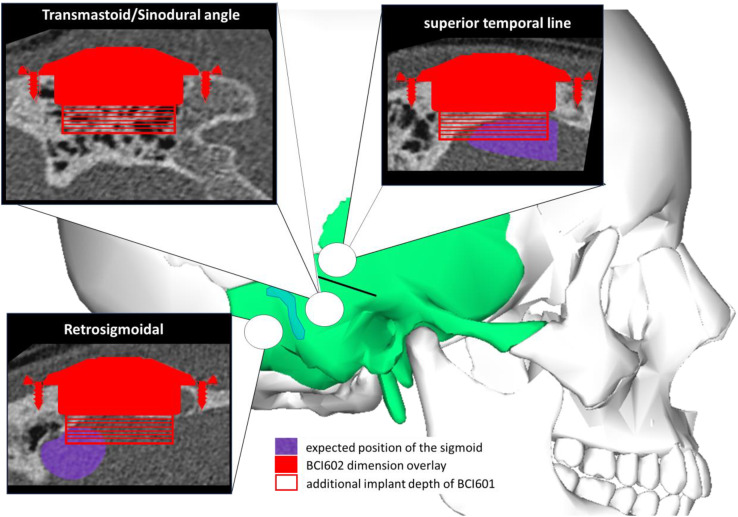
Representative case for surgical planning via OTOPLAN CT images in OTOPLAN showing placement of the BCI602 (red) and additional space required for the BCI601 (broken line) in the three positions as indicated in the picture: (a) retrosigmoidal, (b) transmastoid/sinodural angle, (c) superior to temporal line outlined in an anatomical sketch. CT images are from subject BCI602_13. Positions (a) and (b) would require usage of lifts in the particular case.

OTOPLAN is a surgical planning software that can be used either in the tablet version or on a desktop-PC. The software is intended for use in cochlear implantation and allows CT imaging-based reconstructions to obtain an accurate cochlear lumen view. OTOPLAN estimates cochlear turn length. Available Digital Imaging and Communications in Medicine (DICOM) images from patients’ CT scans were used to evaluate skull thickness of the temporal bone. The aim was to examine if and how devices of the two generations could be placed, in which anatomical position, and under which circumstances. Specifically, it was assessed whether additional lifts were needed and whether dura and/or sinus were exposed and/or compressed. We used the OTOPLAN Electrode Visualization Tool to obtain a more accurate view of the temporal bone in three different possible placement positions: (1) sinodural or transmastoidal position, (2) retrosigmoidal position, or (3) superior to temporal line position. Fig 1 shows an example of this planning process using the OTOPLAN software. All subjects already had been implanted at the time of investigation via the transmastoidal approach.

### Audiological evaluations

All audiometric tests were performed preoperatively (pre-OP) and 3 months after surgery (post-OP) in a soundproof audiometric booth using the AC40E audiometer by Interacoustics (Middelfart, Denmark). Sound-field audiometry (SFA), speech recognition threshold in quiet (SRT), and speech recognition in noise test (SPRINT) at 65 dB sound pressure level in a multi-talker babble were performed. The contralateral ear was masked with narrowband noise during pure-tone and sound field audiometry and with broadband noise during the speech tests. Considering these test circumstances, the two SSD subjects were excluded from the audiological evaluation.

### Hearing-related questionnaire

The Speech, Spatial, and Qualities of Hearing Scale (SSQ 12) questionnaire measures the auditory disability across three domains, namely speech understanding, spatial hearing, and other qualities of hearing, doing so on visual analogue scales ranging from 1 to 10 [11–12]. The questionnaire was translated from English into Czech, the native language of the study cohort, and applied in several studies for validation purposes [3,13–16]. The questionnaire was completed by each child together with its parents to assess the child’s subjective hearing ability before and after implantation (pre-OP vs. post-OP).

### Quality of life assessment

The Assessment of Quality of Life 6 Dimensions (AQoL-6D) questionnaire is comprised of 20 questions covering several aspects affecting quality of life (e.g., the ability to communicate, relationships with family and friends, mental health, physical pain, and others) [17–18]. The questionnaire was translated from English into Czech and applied in several studies for validation purposes [3,13–16]. The questionnaire was completed by each child together with the parents to assess the child’s individual quality of life status pre-OP vs post-OP. Because each individual and the respective time point (pre-OP or post-OP) serves as its own control, even an unvalidated questionnaire would represent a valid comparison of outcomes.

### Data analysis

Descriptive statistics, including mean, standard deviation (SD), median, and range were used to report demographics (e.g., age and gender), baseline characteristics (e.g., etiology), and placement possibilities. The nonparametrically distributed outcomes were analyzed using GraphPad Prism 9.4 statistical software. The Wilcoxon signed-rank test was applied to evaluate significant differences between unaided and BCI-aided sound-field, SRT, and SPRINT outcomes. Scores from the SSQ12 and AQoL-6D questionnaires were analyzed using the one-sample Wilcoxon signed-rank test to test for statistical significance. Speech as well as questionnaire outcomes are summarized in boxplots with the vertical margins of a box representing the upper and lower quartiles (interquartile range), the vertical line inside the box marking the median, and the whiskers extending from the highest to lowest observation.

### Surgical procedure step by step

Anesthesia and Positioning: The procedure is usually performed under general anesthesia.

Position the patient supine with the head turned contralaterally to expose the mastoid and retroauricular area. Incision and Soft Tissue Dissection: Make a postauricular or retroauricular incision approximately 5–6 cm in length. Carefully elevate the periosteum and soft tissues to expose the mastoid cortex while preserving the skin and subcutaneous tissue (we generally used 2 flaps technique, first flap is composed of skin and soft tissues, second flap is periostal flap). Creation of the Bone Bed: Using a high-speed drill, create a bony well (bed) to accommodate the floating mass transducer (FMT) of the Bonebridge implant. The depth and size of the bed depend on the implant model. Take care to avoid injury to the sigmoid sinus and dura mater during drilling. Implant Placement: Place the Bonebridge implant’s FMT into the prepared bone bed ensuring stable, flush seating without tension on surrounding tissues. Secure the implant with screws provided in the surgical kit.

Routing of the Cable: Position the implant’s coil and connecting cable subperiosteally, ensuring they lie flat without kinking. Create a subcutaneous tunnel for the coil receiver placement. Wound Closure: Reposition the soft tissues and periosteum over the implant.

Close the incision in layers using absorbable sutures for the deep layers and skin sutures or staples for the skin.

Postoperative Care: Apply a sterile dressing. Monitor for signs of infection or hematoma.

Schedule activation of the external audio processor typically 3–6 weeks after surgery once healing is confirmed.

## Results

The study cohort included 21 pediatric patients, 19 of whom were suffering from CHL and 2 with SSD. In the CHL group, a majority of 15 patients had aural atresia, 2 suffered from chronic otitis media, 1 had otosclerosis, and 1 had received the implant due to a congenital middle ear malformation. Five children were implanted with the BCI601 and 16 received the BCI602. One each of the two SSD subjects received a BCI601 or BCI602. The study cohort comprised 10 females and 11 males. Age at implantation was 10.7 ± 4.1 years, ranging from the youngest at 6 years up to 19 years of age. Detailed demographic and surgical information are summarized in Table 1.

**Table 1:** Patient demographics and surgical details.

Patient ID	Age	Sex	Implant side	PTA ipsi		PTA contra		Etiology	Use of lift	Exposure of dura mater?	Compression of sigmoid sinus?	Complication/Revision
				AC	BC						AC	BC
BCI601_01	11	M	R	59	10	60	9	Atresia bilat.	no	no	no	–
BCI601_02	14	F	L	58	10	20	8	Atresia l.sin.	1 mm	no	no	–
BCI601_03	16	F	R	59	10	18	10	Atresia l.dx.	no	no	yes	1 year post-op: retroauricular emphysema *(fat/fibrin glue insertion)*
BCI601_04	11	F	L	60	20	45	20	Atresia bilat.	no	yes	no	–
BCI601_05	12	F	R	110	80	10	0	SSD l.dx.	no	no	no	–
BCI602_01	6	F	L	58	11	46	10	Atresia l.dx.	no	no	no	–
BCI602_02	19	M	L	101	85	21	15	SSD l.sin.	no	no	no	–
BCI602_03	9	M	L	54	8	20	5	Atresia l.sin.	no	no	no	–
BCI602_04	10	M	R	68	8	13	8	Atresia l.dx.	no	yes	no	–
BCI602_05	13	M	R	69	16	86	27	OMCHS bilat.	no	no	no	–
BCI602_06	8	F	L	64	14	64	16	Atresia bilat.	no	no	no	–
BCI602_07	15	M	L	60	8	16	9	Atresia l.sin.	no	no	no	–
BCI602_08	16	F	R	49	8	65	15	bilat. congenital ME malformation	no	no	no	–
BCI602_09	9	F	L	74	28	89	78	OMCHS bilat.	no	yes	no	–
BCI602_10	6	M	R	68	13	66	16	Atresia l.sin.	no	yes	no	–
BCI602_11	17	F	L	58	15	14	11	Otosclerosis l.sin.	no	no	no	–
BCI602_12	7	M	R	65	9	66	9	Atresia bilat.	no	no	no	–
BCI602_13	6	M	L	65	14	65	15	Atresia bilat.	no	no	no	1,5 years post-op: head trauma *(explantation for discomfort)*
BCI602_14	7	M	L	65	5	5	5	Atresia l.sin.		yes	no	–
BCI602_15	6	F	L	65	14	66	14	Atresia bilat.	no	no	no	–
BCI602_16	6	M	L	55	5	8	5	Atresia l.sin.	no	no	no	3 months post-op: head trauma *(aspiration of subcut. hematoma)*

M, male; F, female; L, left; R, right; SSD, single-sided deafness; ME, middle ear; sin/L, left; dx/R, right; bilat, bilateral; OMCHS, otitis media chronica secretorica.

### Surgical outcomes and complications

Before implanting the BCI601, optimal placement of the FMT was planned via pre-OP CT scans of the temporal bone. The transmastoidal approach was carried out in all patients. The BCI lift product was used only in one patient implanted with a BCI601. The dura was exposed in 1 patient with a BCI601 and in 4 patients receiving a BCI602. The sigmoid sinus was compressed in 1 patient receiving the first-generation Bonebridge. No complications occurred during surgery. Three minor complications occurred in the BCI602 sub-cohort: one child suffered on the first day after surgery from torticollis, which was resolved via sternocleidomastoid muscle release (the so-called Bemmer procedure – a form of electrotherapy that uses low-frequency electrical currents to stimulate nerves and muscles. It’s sometimes used in rehabilitation medicine, physiotherapy, or pain management.); another developed a cough and was treated with an over-the-counter cough syrup containing butamirate (a cough suppressant) and guaifenesin (an expectorant); a third subject suffered acute otitis media on the 4th day post-op and this was resolved by antibiotic treatment. Late complications occurred in 3 cases. Three months post-op 1 child suffered a head trauma and the resulting subcutaneous hematoma was resolved via aspiration. One year post-op, retroauricular emphysema was reported in 1 case. This was resolved by insertion of fat and fibrin glue around the FMT. Another child suffered a head trauma 1.5 years later and the BCI602 had to be explanted due to discomfort. No patient reported pain or irritation of the skin at or around the implant site.

### Post-operative surgical planning using OTOPLAN

**Fig 2:**
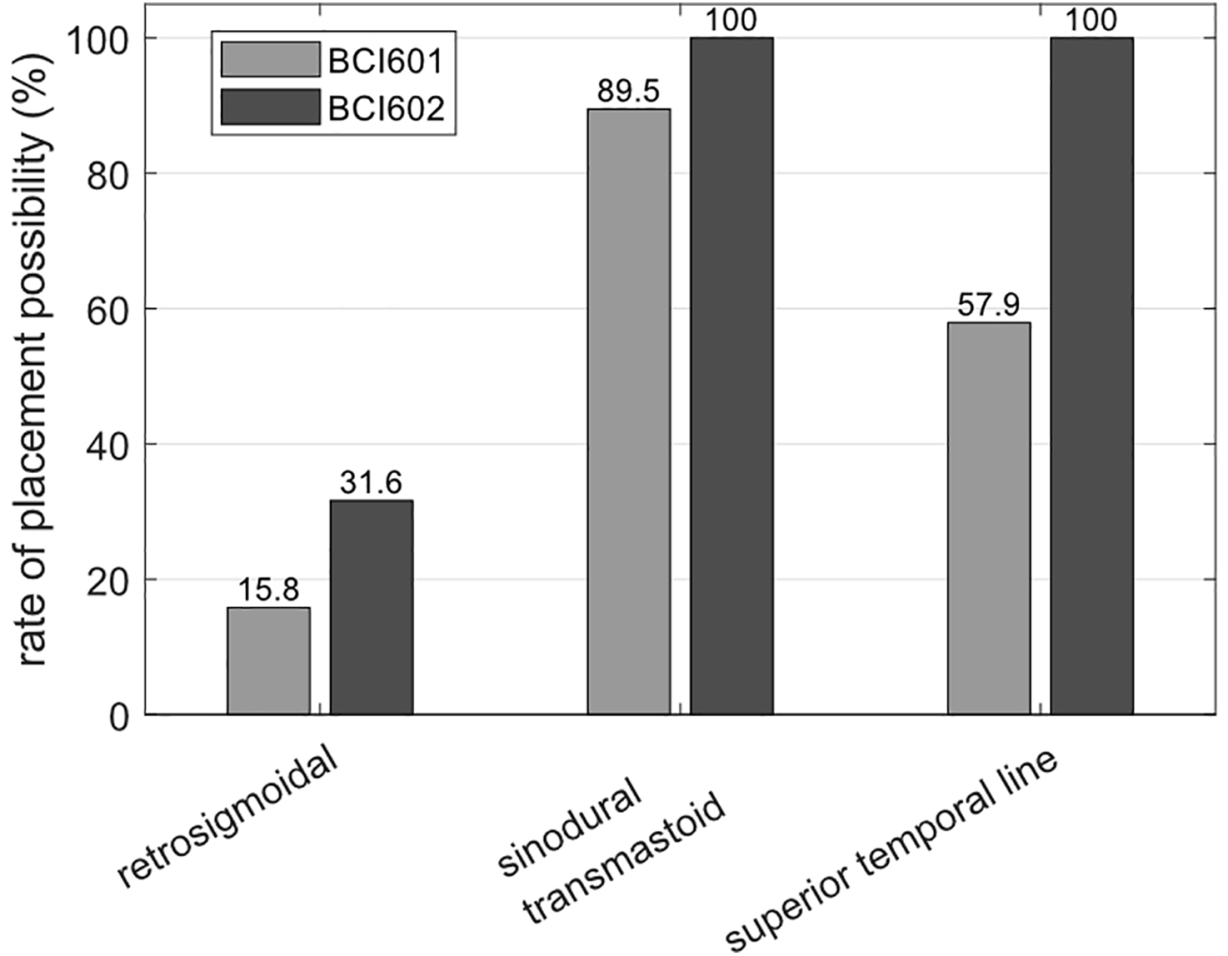
Percentage of possible placement at three different locations for the BCI601 and BCI602 variants, respectively, based on the imaging data available for the cohort.

Preoperative CT images of 19 subjects were available. Although the Bonebridge devices were implanted successfully in all subjects in the transmastoidal position, we investigated post-operatively the theoretical placement possibilities with both BCI601 and BCI602, including any necessity for using of lifts and the expected dura and/or sinus involvement or compression. Fig 2 shows the frequency of different placement options for the cohort. Tables 2 and 3 outline the resulting findings in detail for the BCI601 and BCI602 implant variants, respectively.

**Table 2:** OTOPLAN planning: Placement possibilities BCI601.

Patient ID	Retrosigmoidal possible?	Lifts required?	Compression as per planning	Sinodural/trans-mastoid possible?	Lifts required?	Compression as per planning	Superior temporal line	Lifts required?	Compression as per planning
BCI601_01	no	NA	NA	yes	no	no	no	NA	NA
BCI601_02	yes	4 mm	no	yes	no	no	yes	4 mm	no
BCI601_03	no	NA	NA	yes	4 mm	no	yes	3 mm	no
BCI601_04	no	NA	NA	yes	4 mm	<1 mm	no	NA	NA
BCI601_05	no	NA	NA	yes	1 mm	no	yes	2 mm	no
BCI602_01	no	NA	NA	no	NA	NA	no	NA	NA
BCI602_02	yes	no	no	yes	no	no	yes	no	no
BCI602_03	no	NA	NA	yes	2 mm	no	yes	4 mm	no
BCI602_04	no	NA	NA	yes	3 mm	no	no	NA	NA
BCI602_05	no	NA	NA	yes	3 mm	no	yes	4 mm	1 mm
BCI602_06	no	NA	NA	yes	3 mm	no	no	NA	NA
BCI602_07	yes	3 mm	no	yes	no	no	yes	4 mm	no
BCI602_08	no	NA	NA	no	NA	NA	no	NA	NA
BCI602_09	no	NA	NA	yes	4 mm	no	no	NA	NA
BCI602_10	no CT data	no CT data	no CT data	no CT data	no CT data	no CT data	no CT data	no CT data	no CT data
BCI602_11	no CT data	no CT data	no CT data	no CT data	no CT data	no CT data	no CT data	no CT data	no CT data
BCI602_12	no	NA	NA	yes	4 mm	no	yes	4 mm	no
BCI602_13	no	NA	NA	yes	2 mm	no	yes	4 mm	1 mm
BCI602_14	no	NA	NA	yes	3 mm	no	yes	4 mm	1 mm
BCI602_15	no	NA	NA	yes	4 mm	no	yes	4 mm	1–2 mm
BCI602_16	no	NA	NA	yes	1 mm	no	no	NA	NA

**Table 3:** OTOPLAN planning: Placement possibilities BCI602.

Patient ID	Retrosigmoidal possible?	Lifts required?	Compression as per planning	Sinodural/trans-mastoid possible?	Lifts required?	Compression as per planning	Superior temporal line	Lifts required?	Compression as per planning
BCI601_01	no	NA	NA	yes	no	no	yes	1 mm	2 mm
BCI601_02	yes	no	no	yes	no	no	yes	no	no
BCI601_03	yes	1 mm	1 mm	yes	no	no	yes	no	no
BCI601_04	no	NA	NA	yes	no	no	yes	1 mm	<1 mm
BCI601_05	yes	1 mm	no	yes	no	no	yes	no	no
BCI602_01	no	NA	NA	yes	yes	no	yes	1 mm	no
BCI602_02	yes	no	no	yes	no	no	yes	no	no
BCI602_03	no	NA	NA	yes	no	no	yes	no	no
BCI602_04	no	NA	NA	yes	no	no	yes	1 mm	<1 mm sinus
BCI602_05	yes	1 mm	no	yes	no	no	yes	1 mm	no
BCI602_06	no	NA	NA	yes	no	no	yes	1 mm	notransma
BCI602_07	yes	no	no	yes	no	no	yes	no	no
BCI602_08	no	NA	NA	yes	no	<0.1	yes	1 mm	no
BCI602_09	no	NA	NA	yes	no	no	yes	1 mm	<1 mm
BCI602_10	no CT data	no CT data	no CT data	no CT data	no CT data	no CT data	no CT data	no CT data	no CT data
BCI602_11	no CT data	no CT data	no CT data	no CT data	no CT data	no CT data	no CT data	no CT data	no CT data
BCI602_12	yes	1 mm	~2 mm	yes	no	no	yes	no	no
BCI602_13	no	NA	NA	yes	no	no	yes	1 mm	no
BCI602_14	no	NA	NA	yes	no	no	yes	1 mm	no
BCI602_15	no	NA	NA	yes	no	no	yes	1 mm	<1 mm
BCI602_16	no	NA	NA	yes	no	no	yes	1 mm	>2 mm sinus

Out of the 19 children, placement of the BCI601 in the retrosigmoid position was feasible in 3 cases (16%) and would not have been possible due to size limitations in the remaining 16 cases (84%). Of those 3 subjects, 1 placement in the retrosigmoid plane would have been possible without the use of lifts and compression, 1 would have required a 4 mm lift, and 1 a 3 mm lift. Placement of the BCI601 in the sinodural angle was possible in 17 cases (89%), and only 2 subjects (11%) had anatomical features not allowing the implantation. Four sinodural placements were possible without the use of lifts, 4 cases would have required a 4 mm lift, and 4 a 3 mm lift. In 2 cases a 2 mm lift was suggested, and 2 children required 1 mm lifts. Out of the 19 children, the BCI601 planning revealed that in 11 cases a superior to temporal line placement would have been possible (58%) and no implantation would have been possible in this plane in 8 cases (42%). Most of the cases (*n* = 8) would require 4 mm lifts and 2 cases would require 2 mm and 3 mm lifts, respectively. Planning revealed compression of as much as 1 mm of the underlying structures in 3 cases.

Investigating the new generation Bonebridge, the BCI602, which is about half the size in terms of implantation depth, revealed a possible placement in the retrosigmoid plane in 7 cases (37%) and in 12 patients placement would not be possible (63%). In 3 of those cases, no lifts and no compression were to be expected. The remaining 4 would require 1 mm lifts and would include in 2 of those cases compression of about 1 mm or 2 mm. The BCI602 placement was theoretically (and practically) possible in 100% of cases. According to planning, and as was noted in the surgical records, the use of lifts was necessary in 1 case and 1 case was expected to have slight compression of less than 0.1 mm. Placing the BCI602 would have been possible in the superior to temporal line in 100% of the children while using 1 mm lifts in 11 cases (58%) and with an expected compression between 1 mm and 2 mm of sinus and/or dura in 6 cases. Despite the possibility for placement, in 2 cases this position was not recommended as placement because it would have been less than ideal due to audio-processor positioning and curvature and/or sharp edges of the implant that would be prone to possible mechanical damage.

### Audiologic assessment

**Fig 3:**
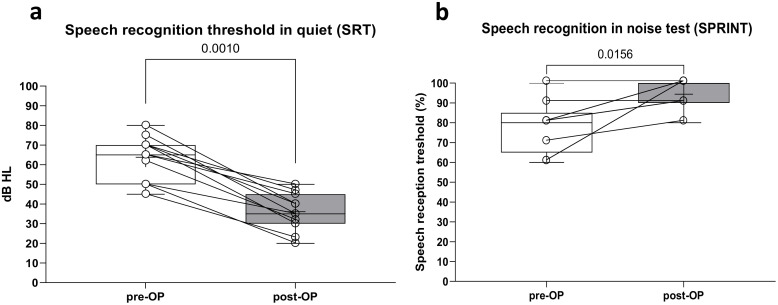
Audiological outcomes for the cohort while excluding the two cases of SSD; (a) shows the aided and unaided sound field thresholds, shaded areas denote standard deviation; (b) shows speech reception thresholds in quiet (dB SPL); and (c) shows results of SPRINT test in noise (%). White boxes represent the pre-operative and gray boxes the post-op/aided condition: mean, median, and SD. Circles show individual improvement.

No significant difference was found in any of the audiological measurements for the two device generations. Outcomes data were therefore pooled in order to achieve greater statistical strength and are not displayed separately. The mean preoperative SRT outcome was 63.82 ± 11.15 dB and improved to a mean of 36.09 ± 9.54 dB after implantation (*p* = 0.0010) (The SPRINT test yielded a mean preoperative score of 77.78% ± 13.02% and improved to 94.44% ± 7.27% after implantation (**p* *= 0.0156; Fig 3). The pre-OP sound field pure tone average (PTA4) was 55.79 ± 10.04 decibels hearing level (dB HL) before implantation and improved to 23.36 ± 3.39 dB HL after Bonebridge provision. Fig 3 summarizes the audiological results.

### Results of the subjective questionnaire

**Fig 4:**
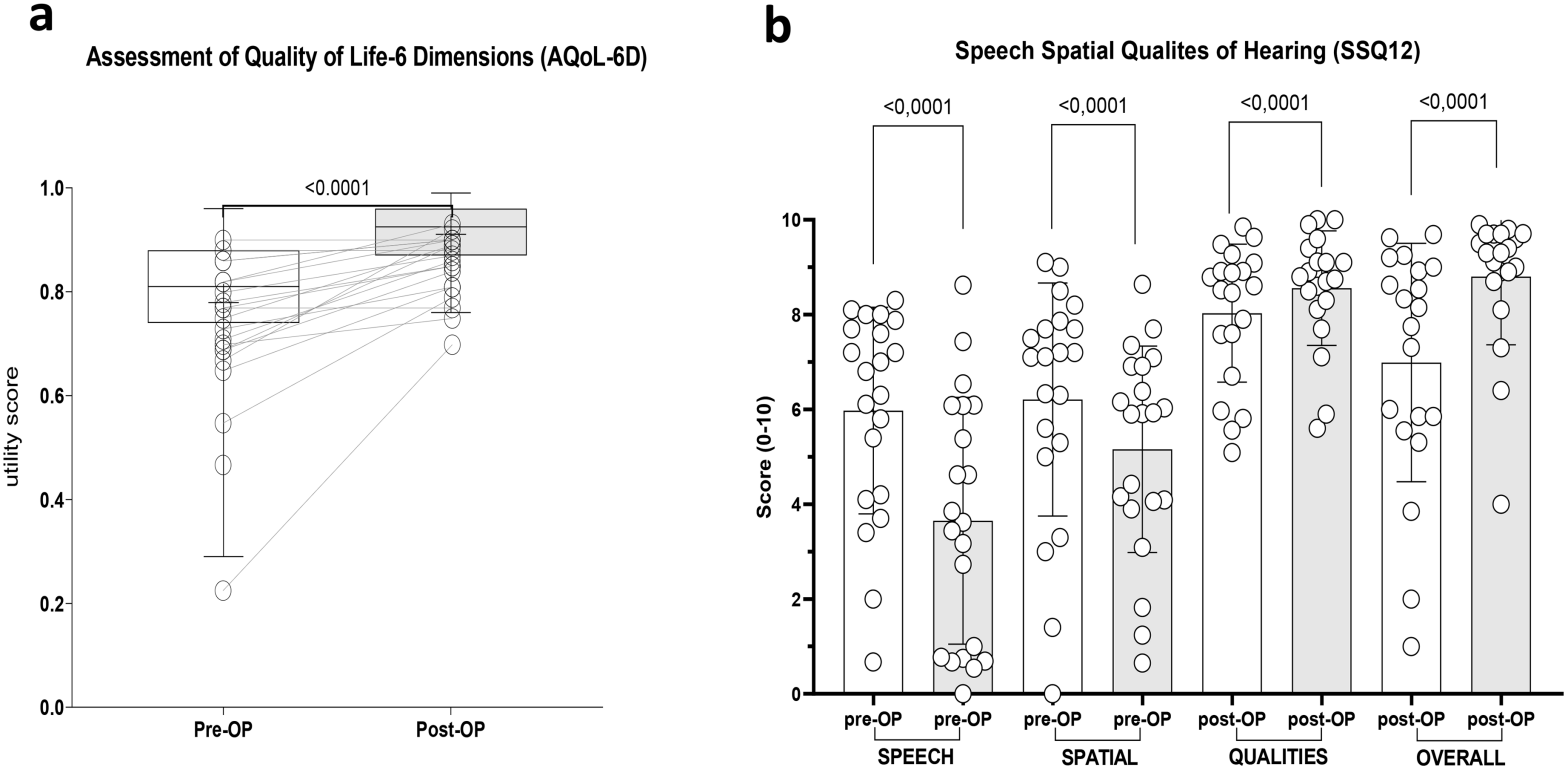
Questionnaire results. (a) AQoL-6D Utility scores comparing pre-OP and post-OP scores. Horizontal lines denote the median. (b) SSQ12: Sub‐scores of speech, spatial hearing, and perceptive qualities as well as the total score comparing pre-OP and post-OP results. There was a highly statistically significant improvement in each sub-score. Circles show individual data points.

Patients with SSD, too, were included for the questionnaire results. The weighted mean utility of the AQoL-6D questionnaire increased significantly, from 0.78 ± 0.15 before implantation to 0.91 ± 0.06 after implantation (*p* < 0.0001). All dimensions of the SSQ12 questionnaire (Speech, Spatial, Qualities of Hearing, and overall score) revealed highly significant subjective benefit (**p <* *0.0001). The dimension describing speech understanding improved from 6.0 ± 2.2 to 8.6 ± 1.2. Questions about localization and spatial hearing improved from 3.7 ± 2.6 to 7.0 ± 2.5. Investigating the quality of hearing revealed mean preoperative outcomes of 6.2 ± 2.5, which improved to 8.8 ± 1.4. The mean overall score improved from 5.2 ± 2.2 before implantation to 8.0 ± 1.5 after the surgery. Fig 4 illustrates findings of the subjective questionnaire.

## Discussion

The primary goal of this study was to evaluate placement possibilities in children for the new generation of the Bonebridge implant, the BCI602, and to compare it to its precursor model, the BCI601. Additionally, the audiological benefits, quality of life, and safety in children implanted with the Bonebridge implant system were investigated. First, it was assessed whether the smaller footprint of the new BCI602 implant would extend surgical placement options and therefore, in theory, broaden the group of potential implant candidates. To answer this question, we used the surgical planning software OTOPLAN to perform the preoperative planning for both the BCI601 and BCI602 in actual non-adult Bonebridge implant recipients from our clinic.

Typically, the ideal location is on the mastoid bone, 50–55 mm from the ear canal, and with the indicator in line with, but not touching, the top of the pinna [19,20]. Bone thickness may be a limiting factor in children, as bone frequently is thinner than the implant bed that is required. The bone at the insertion site should ideally have thickness of 3–4 mm to support the implant, although accommodations can be made for bone thickness < 3 mm (even thinner is possible with the BCI602 and the use of lifts). The thickness and curvature of the skull of non-syndromic children is typically not a concern, especially after the age of 5 years, but in syndromic children (e.g., Treacher-Collins syndrome and CHARGE [coloboma, heart defects, atresia (choanal), restricted development (mental), genital hypoplasia, and ear abnormalities] syndrome) these characteristics are less consistent and therefore less reliable. Nonetheless, our experience with the BCI602 in such cases is still very positive in terms of both surgery and post-operative patient satisfaction [3,13,14]. Preoperative planning is supported by Bonebridge’s manufacturer in difficult cases, whereas implantation of the device is not a difficult task for otologists in cases of normal temporal bones and, in these cases, preoperative planning is typically not required. On the other hand, patients with congenital malformations and difficult anatomies and/or history of multiple middle ear surgeries or young individuals with limited bone thickness are slightly more challenging when it comes to securing the FMT without penetrating the bony well to either the middle and posterior cranial fossa or sigmoid sinus. Our present investigation showed that placement of the BCI602 in the sinodural/transmastoidal region based on OTOPLAN planning was possible in all cases. This was also confirmed in practice, inasmuch as all 19 children were implanted by this approach and with no complications. The dura was exposed in five cases but this had no impact on either safety or the audiological performance and patient satisfaction, according to the clinical records.

The tests chosen for audiological assessment aimed to reflect real-life benefit and were complemented by the subjective evaluation of QoL and quality of hearing via questionnaires. Numerous studies have reported beneficial audiological outcomes and, therefore, high patient satisfaction, accompanied by low rates of complications [1,3,14,15,19]. This is in line with our experience and data presented in this work.

It is gratifying to observe that our audiological measures go hand in hand with the subjective self-assessment reported by patients and their parents. Outcomes reported in the literature from children reached an average aided sound field threshold close to normal hearing with the BCI601 (i.e., 24 dB HL for 67 implants [21–23]), which is similar to our observations (23.36 ± 3.39 dB HL and in the SSD: 22.01 ± 5.57). Magele et al. reported in a meta-analysis based on six studies in children with CHL or mixed hearing loss an average functional gain of 34 dB [1,24–26]. The average functional gain in our population was a very similar 32.4 dB. The investigation here of SRT resulted in significant benefit of 30.26 ± 10.07 dB HL and the measured SPRINT benefit was almost 20%. Zernotti et al. had investigated 14 congenital atresia patients implanted with the BCI601, which at the time was the only model available, and reported significant improvements in hearing thresholds and word recognition scores accompanied by low complication rates. In our study cohort, out of the 21 investigated subjects 3 experienced complications between 3 and 15 months after implantation. All three events could be resolved and were not related to the device itself, rather they were caused by head trauma resulting from children’s usual behaviors. We were very pleased with the results of the audiological assessment in conjunction with the subjective impressions of the young patients themselves. Analyzing data from the SSQ12 questionnaire revealed statistically significant subjective hearing improvement across all measured dimensions – speech, spatial, qualities of hearing, and hence overall hearing-related QoL – after Bonebridge implantation in children with CHL or SSD. The first generation, BCI601, has already demonstrated lower incidence of skin complications in comparison with other bone-anchored hearing devices [1,23,27,28] and this is expected to be similar or even better in the new-generation BCI602 due to its reduced size.

As compared to the previous model BCI601, according to the manufacturer, the new BCI602 requires nearly 50% less drilling depth due to reduction of the FMT thickness from 8.7 mm to 4.5 mm and flexible implant positioning [29]. This is reflected in our comparison of OTOPLAN placement possibilities between the two devices. In the majority of cases, placement of the BCI602 was possible in all anatomical locations, whereas the BCI601 had several limitations and, despite using lifts as thick as 4 mm, in some cases placement was not advisable. In conjunction with the BCI602’s reduced drilling depth, the bendable transition between the FMT and the coil further opens up new possibilities for difficult anatomies. Implantation of children younger than 5 years of age can be feasible. The transition can also bend medially by as much as 30° in either lateral direction. Again, this improves flexibility in placing the implant, thereby enabling optimal outcomes across a wide range of anatomies and underlying pathologies. The main surgical approach is transmastoid implantation, but in unfavorable anatomical conditions a retrosigmoid approach is chosen [30]. Another option is a middle fossa approach [1,31]. So far, only a single study from Canada in 2020 shows there to be no significant audiological difference between the FMT implanted in the transmastoid or retrosigmoid plane, as well as among using different types of cortical fixation screws and lifts [31]. Our study aimed solely to show the improved surgical possibilities and the fact that the new generation FMT can be placed in almost all anatomical conditions without a need for pre-surgical planning. Nevertheless, we routinely check in the pre-op CT images of the skull area to identify good scull thickness for best placement.

Most reports in the literature related to the Bonebridge implant in children have employed small study groups with at most 20 patients. Our report uses a similar sample size (*n* = 21), which we regard as a limitation of the study, and so further investigations with larger numbers of subjects should be employed in the future.

## Conclusion

Possible surgical placement options for the BCI601 and BCI602 variants of the active transcutaneous hearing system were evaluated using pre-operative imaging data in a group of 21 children who had earlier received Bonebridge implants. Audiological performance, subjective benefit regarding hearing outcome, and quality of life also were assessed. Based on the combination of easy placement and straight-forward surgery, the low complication rate, and the significantly improved objective as well as subjective hearing outcomes, we regard the Bonebridge to be a valuable rehabilitation option for children suffering from CHL or SSD. At the same time, the updated BCI602 version allows for more placement options due to its considerably smaller required drill depth.
